# Factors Involved in the Persistence of a Shiga Toxin-Producing *Escherichia coli* O157:H7 Strain in Bovine Feces and Gastro-Intestinal Content

**DOI:** 10.3389/fmicb.2018.00375

**Published:** 2018-03-09

**Authors:** Audrey Segura, Pauline Auffret, Delphine Bibbal, Marine Bertoni, Alexandra Durand, Grégory Jubelin, Monique Kérourédan, Hubert Brugère, Yolande Bertin, Evelyne Forano

**Affiliations:** ^1^Institut National de la Recherche Agronomique, UMR-MEDIS, Université Clermont Auvergne, Clermont-Ferrand, France; ^2^IRSD, Institut National de la Santé Et de la Recherche Médicale, Institut National de la Recherche Agronomique, ENVT, UPS, Université de Toulouse, Toulouse, France

**Keywords:** *Escherichia coli*, STEC, EHEC, O157:H7, biofilms, stress response, persistence

## Abstract

Healthy cattle are the primary reservoir for O157:H7 Shiga toxin-producing *E. coli* responsible for human food-borne infections. Because farm environment acts as a source of cattle contamination, it is important to better understand the factors controlling the persistence of *E. coli* O157:H7 outside the bovine gut. The *E. coli* O157:H7 strain MC2, identified as a persistent strain in French farms, possessed the characteristics required to cause human infections and genetic markers associated with clinical O157:H7 isolates. Therefore, the capacity of *E. coli* MC2 to survive during its transit through the bovine gastro-intestinal tract (GIT) and to respond to stresses potentially encountered in extra-intestinal environments was analyzed. *E. coli* MC2 survived in rumen fluids, grew in the content of posterior digestive compartments and survived in bovine feces at 15°C predicting a successful transit of the bacteria along the bovine GIT and its persistence outside the bovine intestine. *E. coli* MC2 possessed the genetic information encoding 14 adherence systems including adhesins with properties related to colonization of the bovine intestine (F9 fimbriae, EhaA and EspP autotransporters, HCP pilus, FdeC adhesin) reflecting the capacity of the bacteria to colonize different segments of the bovine GIT. *E. coli* MC2 was also a strong biofilm producer when incubated in fecal samples at low temperature and had a greater ability to form biofilms than the bovine commensal *E. coli* strain BG1. Furthermore, in contrast to BG1, *E. coli* MC2 responded to temperature stresses by inducing the genes *cspA* and *htrA* during its survival in bovine feces at 15°C. *E. coli* MC2 also activated genes that are part of the GhoT/GhoS, HicA/HicB and EcnB/EcnA toxin/antitoxin systems involved in the response of *E. coli* to nutrient starvation and chemical stresses. In summary, the large number of colonization factors known to bind to intestinal epithelium and to biotic or abiotic surfaces, the capacity to produce biofilms and to activate stress fitness genes in bovine feces could explain the persistence of *E. coli* MC2 in the farm environment.

## Introduction

Enterohaemorrhagic *E. coli* (EHEC) of serotype O157:H7 is a highly pathogenic subgroup of Stx-producing *E. coli* (STEC) with food-borne etiology responsible for severe clinical symptoms such as hemorrhagic colitis or hemolytic uremic syndrome (HUS) (Nataro and Kaper, 1998). The production of Shiga toxin (Stx) and intestinal attaching and effacing (A/E) lesions are the most important virulence attributes of these pathogens (Karmali, 1989; Nataro and Kaper, 1998). Ruminants are the principal reservoir of *E. coli* O157:H7 and fecal shedding is the main source of contamination of food and farm environments (Nataro and Kaper, 1998). Consequently, cattle play a significant role in the epidemics of human O157:H7 infections since outbreaks are mainly associated with the consumption of contaminated bovine food products (meat, raw milk, dairy products) as well as fruits or vegetables contaminated via irrigation water or ruminant manure (Karmali, 1989; Nataro and Kaper, 1998; Persad and LeJeune, 2014).

Farm environment acts as a source of cattle contamination. Colonization of cattle with *E. coli* O157:H7 is typically transient and the concentration and frequency of *E. coli* O157:H7 shedding vary among individual hosts. For example, cattle “super-shedder” (>more than 10^4^ CFU *E. coli*/g of feces) is responsible for the presence of the majority of *E. coli* O157:H7 in the farm environment (Munns et al., 2016). Consequently, a decrease in *E. coli* O157:H7 shedding at the farm level is likely one of the most cost-effective and safe intervention strategies to prevent further food chain contamination (Soon et al., 2011). Several host and farm practices have been associated with the increase in *E. coli* O157:H7 prevalence including animal age, season or herd type (Soon et al., 2011; Zhang and Woolhouse, 2011; Jeong et al., 2013; Widgren et al., 2015). Natural transmission of *E. coli* O157:H7 between animals occurs directly through the fecal-oral route or indirectly via the farm environment. *E. coli* O157:H7 can survive for extended periods in animal feces and in the farm environment (e.g., cattle drinking water, animal feed, effluents, soil, plants) contributing to the contamination and recontamination of cattle, and ultimately to human infections (Soon et al., 2011). For example, *E. coli* O157:H7 strains have been isolated over a period of 2 years from dairy herds in the USA, and O157:H7 clones have been isolated in a same farm over a period of at least 17 months in England (Shere et al., 1998; Liebana et al., 2005).

The mammalian intestine is the natural habitat of *E. coli* O157:H7 strains. When exposed to extra-intestinal environments, the bacteria must resist against wildly fluctuating environmental stresses to ensure its survival. The persistence and spreading of *E. coli* O157:H7 within farms can be affected by various factors such as microbial interactions (antagonism, competition for nutrients), physicochemical parameters (pH, temperature, solar radiation, rainfall events), physiological parameters (ability of the bacteria to form biofilms, fitness factors supporting persistence) or farming practices (slurry, manure) (Fremaux et al., 2006; Soon et al., 2011; Jeong et al., 2013; Vogeleer et al., 2015). Overall, persistence and spreading of *E. coli* O157:H7 at the farm level depend on strain type and its ability to survive in the farm environment as well as duration and magnitude of shedding by individual animal (Conedera et al., 2001; Fremaux et al., 2006).

It is important to better understand factors influencing persistence and spreading of *E. coli* O157:H7 at the farm level. In a previous prevalence study, we identified *E. coli* O157:H7 bovine shedders in French slaughterhouses (Bibbal et al., 2015). The farms from which they came from were visited several times in order to detect STEC O157:H7 in cattle. From these sampling campaigns, the isolate *E. coli* O157:H7 MC2 was considered as a persistent strain. In this study, the genome of *E. coli* MC2 was analyzed to i) determine its phylogenetic position among *E. coli* O157:H7 strains and ii) define its virulence properties to estimate the potentiality of the bacteria to cause human illnesses. *E. coli* MC2 was also cultured in different compartments of the bovine gastro-intestinal tract (GIT) to test its capacity to survive during its transit through the bovine gut. The capacity of *E. coli* MC2 to survive and form biofilms in bovine feces was also investigated, and gene expression experiments were performed to analyze the bacterial transcriptional response to stresses potentially encountered outside the animal GIT.

## Materials and methods

### Genome analysis

The MC2 genome was previously sequenced and the draft genome deposited in DDBJ/ENA/GenBank under the accession number NJDB00000000.1 (Auffret et al., 2017).

The predicted coding DNA sequences (CDS) in MC2 genome were screened for virulence factors using blastn (version 2.2.28+) (with minimum coverage and identity thresholds of 70%) and the Virulence Factors database DNA dataset (http://www.mgc.ac.cn/VFs/, release Fri May 27 10:06:02 2016; Chen et al., 2005). The MC2 serotype was confirmed *in silico* using SerotypeFinder (version 1.1; Joensen et al., 2015). The Stx subtype, the occupancy of the *yehV, wrbA, argW*, and *sbcB* loci, and the Stx-associated bacteriophage insertion (SBI) boundary sequences were first analyzed by PCR amplification (Shaikh and Tarr, 2003; Besser et al., 2007; Auvray et al., 2009) and then confirmed by *in silico* analysis. The SBI genotyping nomenclature (Shringi et al., 2012) was used to design the SBI genotype code of MC2. Phylogrouping was performed as previously described (Clermont et al., 2013). Lineage-specific polymorphism (LSPA-6), based on sequence length polymorphism of six loci (Yang et al., 2004), was also analyzed using *in silico* PCR. LSPA-6 genotypes 111111 and 211111 are assigned to lineage I and I/II strains respectively and all deviations from the genotypes 111111 and 211111 were classified as LSPA6 lineage II (Zhang et al., 2007; Ziebell et al., 2008). The sequences of the O157:H7 strains EDL933 (lineage I) and EC4115 (lineage I/II) (CP001164.1) were used as controls (Zhang et al., 2007; Eppinger et al., 2011). *In silico* probe based method was first used to discriminate clades 1, 2, 3, and 8 independently from 4, 5, 6, 7, and 9 as a group as previously described (Riordan et al., 2008). In a second step, the 23 single nucleotide polymorphisms (SNPs) profile (Amigo et al., 2015) was used to compare the genome sequence of MC2 to that of two O157:H7 strains of clade 7 (08-BKT061141 and 09-BKT048303) described by Söderlund et al. (2014). A whole genome phylogenetic analysis based on SNP differences was performed using CSI Phylogeny (version 1.4) (Kaas et al., 2014) from 26 O157:H7 STEC strains isolated from cattle or clinical cases described in Supplementary Table 1. The unrooted circular tree was generated with Seaview version 4.5.4 (Gouy et al., 2010).

### Bacterial strain characteristics and growth conditions

The O157:H7 *E. coli* strain MC2 was isolated from cattle feces in France (Bibbal et al., 2015). A spontaneous rifampicin-resistant mutant of MC2 (MC2 Rif^R^) was isolated by culturing the wild-type strain on Luria Bertani (LB) agar plates containing rifampicin (100 μg mL^−1^). The reference EHEC O157:H7 strain EDL933 and the bovine commensal *E. coli* strain BG1 (Riley et al., 1983; Bertin et al., 2011) and their spontaneous rifampicin-resistant mutants, EDL933 Rif^R^ and BG1 Rif^R^ (Bertin et al., 2013) respectively, were used as controls. The growth patterns of MC2 Rif^R^, EDL933 Rif^R^ and BG1 Rif^R^ were similar to that of parent strains. The enteropathogenic *E. coli* (EPEC) strain E2348/69 and *E. coli* DH5α were used respectively as a positive and negative controls in the fluorescence-actin staining (FAS) test and in the Vero cell cytotoxicity assay. All *E. coli* strains were routinely cultured in LB broth at 37°C with aeration. The standard biosecurity and safety procedures have been used.

### Animal and digestive contents sampling

Three healthy bulls (Permit number: C6334517) were slaughtered in the experimental slaughterhouse of the “Herbipole” (INRA [National Institute for Agricultural Research] Saint-Genès-Champanelle, France), in accordance with the guidelines of the local ethics committee and current INRA ethical guidelines for animal welfare (Slaughterhouse Permit number: 63345001). Digestive contents (DC) (small intestine, rumen, caecum, colon and rectum) were collected from these three bulls and two additional cows were used for the collection of feces samples. All the animals were fed a mixed diet containing hay (20%), maize (40%), and concentrate (40%). After being collected, the DC were immediately brought to the laboratory. The small intestine contents were directly distributed in sterile tubes (FALCON) without any particular attention regarding anaerobiosis. The other intestinal contents were processed under strictly anaerobic conditions (Chaucheyras-Durand et al., 2006). Briefly, rumen contents were strained through four layers of cheesecloth to remove large feed particles and were then processed without dilution. Caecum, colon and rectum contents were diluted 1:1 in reduced potassium phosphate (PP) buffer (50 mM potassium phosphate, resazurin 0.1%, 40 mM Na_2_CO_3_, 3 mM cysteine, pH 7.2). The PP buffer was prepared as follows: the solution was boiled for at least 5 min, cooled under a 100% CO_2_ atmosphere for 20 min and finally reduced by addition of cysteine. Rumen, caecum, colon and rectum samples were distributed under a stream of CO_2_ in sterile O_2_-free CO_2_-satured Hungate tubes to maintain anaerobiosis. Sensitivity of the intestinal microbiota to rifampicin was confirmed by spotting each digestive content on LB and SMAC agar plates supplemented with 100 μg mL^−1^ rifampicin. All these samples, containing the live endogenous microbiota and noted as unfiltered samples, were used for experiments immediately without freezing. Filtered DC samples were obtained by centrifugation (15 min, 10,000 × g) and filtration through a Steritop and then a Stericup system with membrane pore size of 0.45 and 0.22 μm, respectively (Millipore). The sterility of the filtered DC samples was checked by inoculating an aliquot into a LB agar plate. Filtered DC samples were stored at room temperature until use.

### Enumeration of total cultivable bacteria from digestive contents

The endogenous microbiota was enumerated for each DC sample from the three bulls. The facultative anaerobes and Gram-negative bacteria were enumerated by spreading 100 μL of DC samples on G20 solid medium agar (Chassard et al., 2008) and SMAC agar plates, respectively. Plates were incubated overnight at 39°C before counting colony-forming unit (CFU). To enumerate the density of strict anaerobes, 10-fold serial dilutions of DC samples were performed in a liquid dilution medium of Halliwell and Bryant (1963) under CO_2_ flow. Each dilution (1 mL) was inoculated into O_2_-free CO_2_ Hungate tubes using the complete medium (9 mL) described by Leedle and Hespell (1980). Three Hungate tubes inoculated per dilution were then incubated for 1 week at 39°C under strict anaerobiosis. The most probable numbers of anaerobes were determined with the Mc Grady table (Clarke and Owens, 1983). The presented values are the log_10_ mean number of anaerobes mL^−1^ ± the standard error of the mean (SEM).

### Incubation of *E. coli* strains in digestive contents

The spontaneous mutants MC2 Rif^R^, EDL933 Rif^R^ and BG1 Rif^R^ inoculated from a single colony were first incubated in LB broth supplemented with rifampicin (100 μg mL^−1^) for 7 h at 37°C with aeration. The pre-cultures were then 50-fold diluted in filtered DC samples and grown overnight at 39°C without aeration. The next day, the bacterial concentration was monitored spectrophotometrically at an optical density at 600 nm (OD_600_) and adjusted before inoculating ≈10^4^ cells mL^−1^ in unfiltered DC samples (5 mL each). The DC samples were then incubated at 39°C (internal bovine temperature) under strictly anaerobic conditions with gentle shaking (rumen samples) or without shaking (caecum, colon and rectum samples). Small intestine contents were incubated without any particular attention regarding anaerobiosis and without shaking in order to limit the oxygen supply (oxygen-limited conditions). These conditions (temperature, shaking and oxygenation) were chosen to reflect the *in vivo* conditions for each digestive compartment (Chaucheyras-Durand et al., 2010; Bertin et al., 2011). At each time point, unfiltered DC samples were serially diluted 10-fold in phosphate buffered saline (PBS) pH 7.2, spotted on SMAC agar plates supplemented with rifampicin (100 μg mL^−1^) and incubated overnight at 37°C before bacterial enumeration. Each experiment was replicated at least three times with each DC samples collected once on the three animals. The presented values are the log_10_ mean number of CFU mL^−1^ ± SEM.

### Incubation of *E. coli* strains in feces samples and M9 minimal medium

Feces collected from two cows were pooled and diluted 1:1 (w/v) in PBS buffer (pH 7.2). Unfiltered feces were directly distributed in sterile tubes (FALCON) and immediately used for experiments. Sensitivity of the feces microbiota to rifampicin was confirmed as described above. To obtain sterile samples, the feces were centrifuged and filtered as described above for DC samples. Filtered fecal suspensions were stored at +4°C until use. Feces samples (filtered and unfiltered) and M9 minimal medium supplemented with glucose (40 mM), MgSO_4_ (1 mM), CaCl_2_ (0.1 mM) and trace metals (M9-Glc) (5 mL each) were inoculated with ≈10^4^ and 10^7^ bacteria mL^−1^, respectively, and incubated at 15°C in a refrigerated thermostat (RML6 Lauda) without shaking. At each time point, the bacterial concentration was monitored in feces samples diluted in PBS and spotted on SMAC agar plates supplemented with rifampicin (100 μg mL^−1^) (unfiltered feces samples) or without antibiotic (filtered feces samples) as described above. The bacterial concentration in M9-Glc was quantified spectrophotometrically (OD_600_). Each experiment was replicated at least three times with feces samples collected at three independent days. The presented values are the log_10_ mean number of CFU mL^−1^ ± SEM.

### Short chain fatty acids (SCFA) analysis

Short chain fatty acids (SCFAs) concentration was quantified in DC samples before and after incubation. Briefly, the samples were centrifuged (10,000 × g for 10 min), the supernatants were filtered (0.2 μm) and 30 μl of orthophosphoric acid (75%) were added to 1 mL of supernatant. Total SCFA concentrations were determined by gas chromatography performed by AFYREN INVESTMENT (Biopole Clermont Limagne, Saint Beauzire, France). pH was measured using a HI-8424N pH meter (HANNA instruments). The presented values are the mean (± SEM) of independent measures in DC samples from the three animals.

### Vero cell cytotoxicity assay

Vero cells were seeded at 5 × 10^3^ Vero cells per well on 96-well microplates and cultivated for 24 h in Dulbecco's Minimum Essential Medium (1X) containing non-essential amino acids (Gibco®, Life technologies, UK), supplemented with 10% fetal calf serum (FCS) (Gibco®, South America) at 37°C in a 5% CO_2_ atmosphere. Bacteria were successively cultured in LB broth at 37°C overnight and in LB broth supplemented with 200 mM of mitomycin C (to increase the production of Shiga toxin) at 37°C for 16 h. A volume of 2 mL of bacterial culture containing 10^9^ bacteria mL^−1^ adjusted with LB broth was centrifuged at 6,000 × g for 5 min at +4°C. The supernatant was filtered through a 0.2 μm pore-size membrane filter (Millipore, Carrigtwohill, Ireland) and serially diluted (1:4) with PBS (Sigma-Aldrich, St Louis, USA). The interaction was performed by adding 10 μL of each dilution to each well containing Vero cells. After 72 h of interaction at 37°C, the cells were washed three times with Hank's Balanced Salt Solution (Sigma-Aldrich, St Louis, USA), fixed with 3.7% paraformaldehyde for 15 min, and then washed with 100 μL of PBS three times and 100 μL of 0.01 M Tris-HCl, pH 8.5. The fixed cells were then stained with 100 μL of 1% methylene blue in Tris-HCl for 45 min, washed again with 100 μL distilled water and 100 μL of Tris-HCl, and finally dried overnight. The methylene blue was extracted with 100 μL of 0.1 M HCl by shaking. After 15 min of extraction, 75 μL of cells were transferred to new 96-well microplates and the color density of each well was measured by TECAN microplate reader (Infinite M200) at 660 nm. All assays were repeated twice independently.

### Fluorescent-actin staining (FAS) test

Bacteria were inoculated in LB broth and incubated at 37°C overnight. HeLa cells were seeded at 3.7 × 10^4^ cells per well on Lab-Tek 8 chamber slides (Nunc) and incubated for 24 h in the same conditions as those of Vero cells. HeLa cells were washed three times with preheated Hank's Balanced Salt Solution (37°C). The interaction was done in 500 μL of Dulbecco's Modified Eagle's medium buffered with 25 mM HEPES complemented with 5% FCS, with a starting inoculum of 10^2^ bacteria per HeLa cell. After 6 h of interaction at 37°C, the cells were washed six times with Hank's Balanced Salt Solution, fixed with 4% paraformaldehyde in PBS for 20 min at room temperature, and then permeabilized with 0.25% Triton X-100 in PBS for 5 min. Fluorescent-actin was labeled with Rhodamine-phalloidin (Molecular Probes®) in accordance with the manufacturer's instructions.

### Biofilm formation assay

*E. coli* strains inoculated in LB broth (2 mL) from a single colony were incubated for 7 h at 37°C with aeration. The cultures were then diluted (1:50) in filtered feces and M9-Glc, and incubated 24 h at 39°C with gentle shaking (100 rpm). The next day, the cultures were diluted (1:100) in filtered feces and M9-Glc respectively and 200 μL of bacterial cultures were incubated in triplicate in 96-well polystyrene plate (FALCON). For all biofilm assays, the biofilm-positive strain EDL933 was used as positive control, and wells filled with 200 μL sterile feces or M9-Glc were used as blank values. After incubation of the plates during 6 h, 1, 2, and 3 days at 39°C, and 1, 3, and 7 days at 15°C, unattached cells were removed by washing with Tryptone Salt medium (1 g L^−1^ Bacto Trypton and 8.5 g L^−1^ NaCl). Biofilms were fixed with 100% ethanol for 20 min, the plates were dried for 30 min at room temperature, and the biofilms were stained with crystal violet (0.1%) for 10 min. The crystal violet solution was removed, and the biofilms were washed three times with water. The stain was released with 150 μL of 33% acetic acid. Biofilms were quantified by measuring the absorbance at 595 nm (OD) with a microplate reader (TECAN). Blank values were subtracted from values obtained after bacterial incubation. The strains were classified as previously described: strong (OD_595_ > 0.6), medium (0.6 > OD_595_ > 0.3), or weak (0.3 > OD_595_ > 0.1) biofilm formers or as non–biofilm formers (OD_595_ < 0.1) (Vogeleer et al., 2015). The presented values are the mean of three independent experiments ± SEM.

### RNA extraction

RNA samples were collected when the bacterial strains reached the stationary growth phase in filtered feces samples (7 days) and M9-Glc (30 h). Bacterial suspensions were centrifuged (4,500 × g for 20 min), the supernatants were stored at −20°C for further investigations and the bacterial pellets were flash frozen in liquid nitrogen and rapidly stored at −80°C. The next day, the bacterial pellets were resuspended in TE buffer (10 mM Tris-HCl, 1 mM EDTA; pH 8) in 2 mL Graduated Skirted tube with tethered screw cap (BioSpec) containing 600 mg 0.1 mm diameter zirconia/silica beads (Biospec), 1 volume of AquaPhenol™ (pH 4.5) (MP Biomedicals), 1/10 volume of 10% Sodium Dodecyl Sulfate, and 3.5 μL β-mercaptoethanol (PROLABO). The lysis of bacterial cells was performed using a FastPrep®-24 instruments twice for 30 s with a speed of 6 m/s. Total RNA were purified from the bacterial pellet using the Nucleospin® RNA (Marcherey Nagel) according to the manufacturer recommendations and treated twice with 0.5 μL Turbo DNA-free™ kit (Invitrogen) for 30 min at 37°C to remove genomic DNA. To assess DNA contamination, PCR control on each sample was performed using the *tufA* primers (Supplementary Table 2). The RNA concentration was measured using a Nanodrop ND-1000 spectrophotometer (Nanodrop technologies, France) and the quality of RNA was analyzed using an Agilent 2100 Bioanalyser (Agilent technologies, France) (the high quality of each RNA samples was confirmed with 23S/16S rRNA ratio ≈ 2 and a RNA Integrity Numbers ≥8).

### Relative RT-qPCR

One microgram of each RNA sample was reverse transcribed as previously described (Bertin et al., 2011). Quantitative PCR runs were carried out using the Mastercycler ep realplex apparatus (Eppendorf) as previously described (Bertin et al., 2011). Amplification conditions were as follows: 95°C for 15 s, 55°C for 15 s, and 72°C for 20 s. The relative mRNA quantification was performed using primer pairs described in Supplementary Table 2. Triplicate samples were amplified in each case. Data were normalized using the *tufA* gene as an internal control. Results were calculated using the comparative cycle threshold method. The results presented are averages of the results from at least triplicate experiments ± SEM. Genes showing a >2-fold change in expression were considered to be differentially regulated.

### Statistical analysis

Statistical analyses of the results obtained during incubation of *E. coli* strains in bovine digestive contents and biofilm formation experiments were performed using the two-way ANOVA with the Bonferroni *post-hoc* test and the one-way ANOVA with the Tukey *post-hoc* test, respectively.

For RT-qPCR analysis, fold-changes were considered as significantly different at p < 0.05 (Student's *t*-test).

## Results

### Genome analysis

We first analyzed the MC2 genome (i) to identify the genes encoding virulence factors (VFs) and proteins involved in biofilm formation and (ii) to determine the phylogenetic position of *E. coli* MC2 among *E. coli* O157:H7 strains.

#### Virulence factors

A total of 283 VFs encoding genes previously described among pathogenic *E. coli* were identified in the MC2 genome (Supplementary Table 3). In addition to the locus of enterocytes effacement (LEE) and the Stx toxins, these genes encode the components of adherence systems, iron acquisition systems, secretion systems or proteins involved in biofilm formation.

##### Locus of enterocytes effacement

At least 40 genes belonging to the LEE, which encoded proteins required for A/E lesions, were identified in the MC2 genome. The LEE of *E. coli* MC2 encoded intimin (*eae*γ allele), the Tir intimin-receptor (*tir*) involved in intimate attachment of the bacteria to the host cells and the type III secretion system (T3SS) required to inject Tir directly into the cytosol of the host cells. *E. coli* MC2 harbored the *tir* 255T allele.

##### Toxins

*E. coli* MC2 possessed the genetic information coding for both Stx1a and Stx2c toxins (one copy of each gene). The integration site for the *stx1a*- and *stx2c*-bacteriophages in *E. coli* MC2 was identified in *yehV* and *sbcB* loci, respectively (YS12c SBI genotype code). The MC2 genome carried the *Q*_933_ anti-terminator allele that regulates expression of the gene *stx2*. In addition to *stx*, the MC2 genome harbored the genetic information required for the synthesis of EHEC-hemolysin (*hlyABCD*).

##### Adherence and iron acquisition

In addition to intimin, the MC2 genome carried 44 genes coding for the synthesis of 14 adherence systems identified among pathogenic *E. coli* strains (Table 1). Most of these structures (fimbriae or non-fimbriae) are known to adhere *in vitro* to intestinal cell lines (T84, HT29 or Caco-2; Table 1). The MC2 genome also carried genes coding for the production of flagellum (*fli* and *flg* operons), known to possess adhesive properties, and iron acquisition systems (*entABCDEFS, fepABCDEG, fes, chuA*, and *hma*; Supplementary Table 3).

**Table 1:** Adherence systems encoded by the *E. coli* MC2 genome.

**Adherence system**	**Gene or gene cluster**	***In vitro* cell adherence^a^**	**Receptor**	**Biofilm formation**	**References**
Cah (calcium binding antigen 43 homolog)	*cah*	NA^b^	Calcium	Yes	Torres et al., 2002
Curli	*csgABCDEFG*	T84	Matrix and plasma proteins	Yes	McWilliams and Torres, 2014
ECP (*E. coli* common pilus)	*ecpRABCDE*	HT29, Hep-2, HeLa, HTB-4	Arabinosyl residues	Yes	Rendón et al., 2007
EhaA autotransporter	*ehaA*	Primary bovine epithelial cells (terminal rectum)	Unknown	Yes	Wells et al., 2009
EhaB autotranspoter	*ehaB*	NA^b^	Collagen I, Laminin	Yes	Wells et al., 2009
ELF (*E. coli* laminin-binding fimbriae)	*ycbQRST*	HT29, Hep-2, MDBK	Laminin	ND^c^	McWilliams and Torres, 2014
EspP	*espP*	T84, Bovine primary rectal	Unknown	Yes	Puttamreddy et al., 2010
(Extracellular serine protease)		epilthelial cells			Dziva et al., 2007
F9 fimbriae	*Z2200- Z2206*	EBL, HeLa	Bovine fibronectin Galβ1-3GlcNAc	Yes	McWilliams and Torres, 2014
FdeC (EaeH)	*eaeH*	UM-UC-3, Caco-2, CHO, HeLa, Vero	Unknown	Yes	Easton et al., 2014
HCP (Haemorrhagic coli pilus)	*hcpABC*	T84, Caco-2, HeLa, Hep-2, HT-29, MDBK, cow colon explants	Laminin, fibronectin	Yes	Xicohtencatl-Cortes et al., 2007
Iha	*iha*	HeLa, MDKB	Unknown	ND	Tarr et al., 2000
NlpI lipoprotein	*nlpI*	HBMECs, Intestine-407	Unknown	ND	Teng et al., 2010
T1P (Type I pili)	*fimBE, fimAICDFGH*	HeLa, REC, Colonic, and ileal enterocytes	Mannose	Yes	McWilliams and Torres, 2014
UpaG (EhaG) autotransporter	*upaG (ehaG)*	T24	Fibronectin, Laminin	Yes	Totsika et al., 2012

a *Cell lines: Caco-2, human colon carcinoma; CHO, Chinese hamster ovary; EBL, embryonic bovine lung; HBMECs, human brain microvascular endothelial cells; HeLa, human cervix epithelial carcinoma**;** Hep-2, epithelial cells from epidermoid carcinoma of the human larynx; HT29, human colorectal adenocarcinoma; HTB-4, human bladder transitional carcinoma; Intestine-407, human cervical adenocarcinoma, HeLa derivative;, MDBK, Madin-Darby bovine kidney; REC, human B cell lymphoma; T24, human bladder transitional carcinoma; T84, human colonic adenocarcinoma; UM-UC-3, human bladder carcinoma; Vero, kidney epithelial cells from an African green monkey*.

b *No adherence to the cell lines tested*.

c *Not determined*.

##### Secretion systems, effectors and biofilm formation

In addition to the T3SS encoding genes included in the LEE, the MC2 genome shared the genetic information coding for proteins belonging to the secretion systems ETT2, T2SS, and T6SS (Supplementary Table 3). The type II secretion system (T2SS) (*etp* operon) is required for the secretion of the metalloprotease StcE, also encoded by the MC2 genome. *E. coli* MC2 carried the genes encoding (i) the type III secretion system 2 (ETT2) (*etrA, epaOPQS, eprIJK, eivACEFGJ*) and (ii) Hcp (hemolysin co-regulated protein), VgrG (valine-glycine repeat protein G) and additional proteins constituting the type VI secretions system (T6SS) (Supplementary Table 3). In addition, the MC2 genome encoded 22 non-LEE-encoded (Nle) effectors (Supplementary Table 3). As shown in Table 1, the majority of the genes coding for adherence systems in the MC2 genome are known to be involved in biofilm initiation (11 of 14). Additionally, the genes required for the synthesis of exopolysaccharides (colanic acid [*wca* gene cluster], poly-β-1,6-GlcNAc (PGA) [*pga* gene cluster], cellulose [*bcs* gene cluster]) and T6SS present in the MC2 genome are also involved in biofilm formation.

#### Classification, phylogenetic, and genome signature analyses

Phylogenetic grouping (A, B1, B2, C, D, E and F), lineage (I, II, I/II), and clade (1–9) classifications are currently used to analyze the evolution of O157:H7 *E. coli* strains among different hosts (human and animals) and different countries. *In silico* analysis identified MC2 as phylo-group E, lineage II [based on its LSPA-6 genotype (213111)] and clade 7 *E. coli* strain.

**Figure 1:**
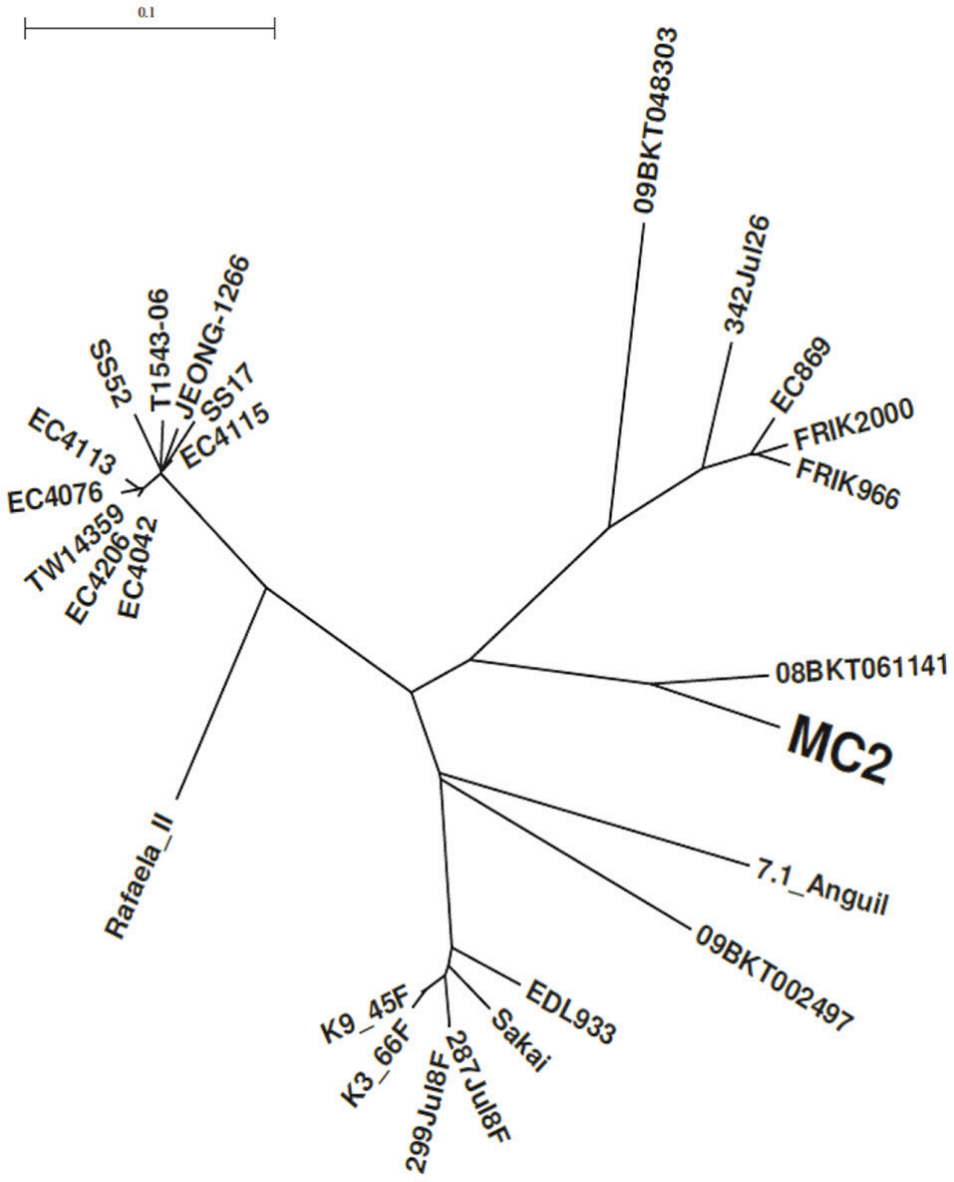
Phylogenetic tree highlighting the position of *E. coli* MC2 relative to other *E. coli* O157:H7 strains. The whole genome SNP based phylogeny was established with CSI phylogeny version 1.4 using EDL933 (AE005174.2) as a reference and standard input parameters (see the Materials and Methods section). The unrooted circular tree was generated with Seaview (version 4.5.4.).

A phylogenetic tree was constructed using a set of 26 genome sequences of *E. coli* O157:H7 strains isolated from cattle and clinical cases (Supplementary Table 1). The tree segregates into three major branches. All the clade 7 bovine strains (*n* = 6) were clustered in the same major branch whereas bovine and human isolates are inter-mixed into the two remaining branches (Figure 1). Consistent with lineage and LSPA-6 classification, the closest relative strain to MC2 was the STEC 08-BKT061141 with a subtype that dominates in Swedish cattle reservoir (Söderlund et al., 2014). As expected, *E. coli* MC2 and 08-BKT061141 possessed identical genetic characteristics (*stx1a* and *stx2c* subtypes, SNPs in *tir*, ECs2357, *fimH* and *agaF* genes, intact *wrbA* site). In depth analysis of the MC2 genome also revealed that premature codon stops in *eutA* and *luxR*, and the absence of deletions in *bcs* and *lsr-tam* operons were absent in the MC2 genome.

### Stx-associated toxicity and A/E lesion formation due to *E. coli* strain MC2

*E. coli* MC2 was tested for the production of toxin Stx (toxicity to Vero cells) and formation of attachment and effacement (A/E) lesions (FAS test), two crucial steps in human EHEC infections. Results analysis showed that the *E. coli* strain MC2 displayed toxicity toward Vero cells (Supplementary Figure 1) and produced A/E lesions on cultured HeLa cells as determined by accumulation of filamentous actin at the sites of adhesion (Supplementary Figure 2).

### Growth or survival of *E. coli* MC2 in bovine digestive contents

**Figure 2:**
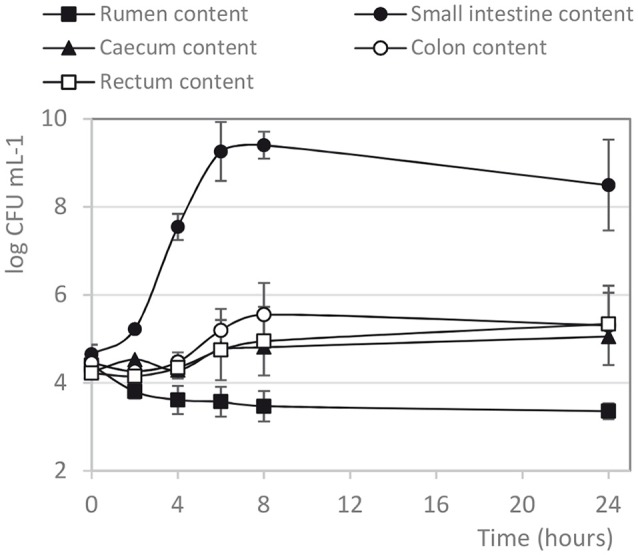
Growth curves of *E. coli* MC2 Rif^R^ incubated in bovine digestive contents. The *E. coli* strain MC2 Rif^R^ was incubated in bovine digestive contents under conditions described in the Materials and Methods section. Each time point is the mean of at least three independent experiments. Statistical analyses were performed with two-way ANOVA with the Bonferroni *post-hoc* test: growth yield in small intestine content was significantly different from the other DC (*p* < 0.001); growth yield in colon and rectum contents was significantly different from that in rumen content (*p* < 0.05).

The capacity of *E. coli* MC2 Rif^R^ to survive through the bovine intestinal tract was analyzed by incubating the bacteria into different digestive contents (DC) collected from rumen, small intestine, caecum, colon and rectum (Figure 2). The spontaneous rifampicin-mutant of the EHEC reference strain EDL933 (control strain) was also included in this study. *E. coli* MC2 Rif^R^ was able to grow in all the digestive compartments except the rumen (Figure 2). In the rumen samples, a 1 log CFU mL^−1^ decrease in the MC2 population was observed after 4 h of incubation and the strain survived for up to 24 h at low level (≈10^3^ CFU mL^−1^) (Figure 2). In contrast, MC2 Rif^R^ grew at high level (5 log CFU mL^−1^ increase) in small intestinal contents, and remained at high concentration up to 24 h. The growth yield of MC2 Rif^R^ in small intestine contents was significantly more important than in the other DC samples (*p* < 0.001; Figure 2). In the other DC, MC2 Rif^R^ population increased from 4 to 8 h and then remained rather stable up to 24 h (≈10^5^ CFU mL^−1^). Similar results were obtained with *E. coli* EDL933 Rif^R^ (data not shown). Differences were observed in the pH value of the different DC at the time of inoculation (T0) (Table 2), with rumen more acidic than the other intestinal compartments. Total SCFAs were also much higher in the rumen than in the lower gut contents before incubation (T0) (Table 2), acetate being the main SCFA present (72.7 ± 7.8 mM acetate, 13.9 ± 1.2 mM propionate and 14.8 ± 2.0 mM butyrate were found in the rumen at T0). After 24 h incubation in the presence of MC2 Rif^R^, the SCFA concentration increased in the DC indicating fermentative activity (data not shown). For example, in the rumen, after 24 h incubation, the concentrations of acetate, propionate and butyrate were 116.2 ± 11.3 mM, 24.1 ± 2.2 mM and 30.1 ± 5.3 mM, respectively.

**Table 2:** Anaerobic bacteria counts, pH values and SCFA concentrations in the digestive contents before incubation (T0).

	**Rumen**	**Small intestine**	**Caecum^a^**	**Colon^a^**	**Rectum^a^**
pH	6.22 ± 0.17	7.29 ± 0.14	6.80 ± 0.08	6.69 ± 0.09	6.68 ± 0.06
Total SCFAs (mM)	105.5 ± 11.3	15.9 ± 4.6	30.3 ± 16.4	15.3 ± 11.8	14.8 ± 11.3
Log_10_ (anaerobes)	10.85 ± 0.19	8.56 ± 0.84	9.24 ± 0.16	9.41 ± 0.24	9.18 ± 0.00

*Values are the mean of measurements in digestive contents from three different animals ± SEM*.

a *These contents were diluted before inoculation (see Materials and Methods)*.

### Survival of *E. coli* MC2 in bovine feces

**Figure 3:**
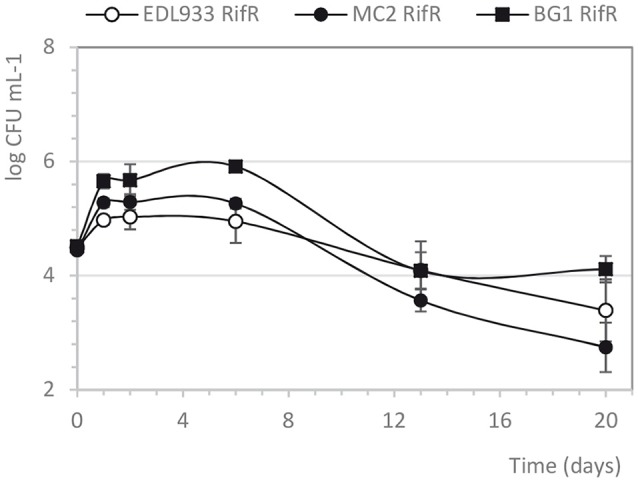
Growth curves of *E. coli* strains incubated in unfiltered bovine feces. Cultures were incubated in feces samples at 15°C without shaking. Each time point is the mean of three independent experiments.

To test the persistence of *E. coli* MC2 outside the animal digestive ecosystem, we first analyzed the growth of MC2 Rif^R^ in feces samples during incubation at 15°C (inoculation at ≈ 4 log CFU mL^−1^). The growth of EDL933 Rif^R^ and the rifampicin-resistant mutant of the bovine commensal *E. coli* BG1 was also analyzed in the same conditions. The average concentration of the endogenous fecal microbiota was ≈10^9^ CFU mL^−1^ at the start of the study. As shown in Figure 3, the bacterial concentration increased slightly during the first day of incubation (5–6 log CFU mL^−1^ were enumerated up to 6 days post-inoculation for the three strains tested) and then decreased to reach ≈10^3^−10^4^ CFU mL^−1^ after 3 weeks of incubation. The growth of MC2 Rif^R^ was very similar to that of *E. coli* EDL933 Rif^R^ (Figure 3). The commensal *E. coli* BG1 Rif^R^ showed slightly higher concentration all along the incubation (Figure 3).

### Biofilm formation in bovine feces

**Figure 4:**
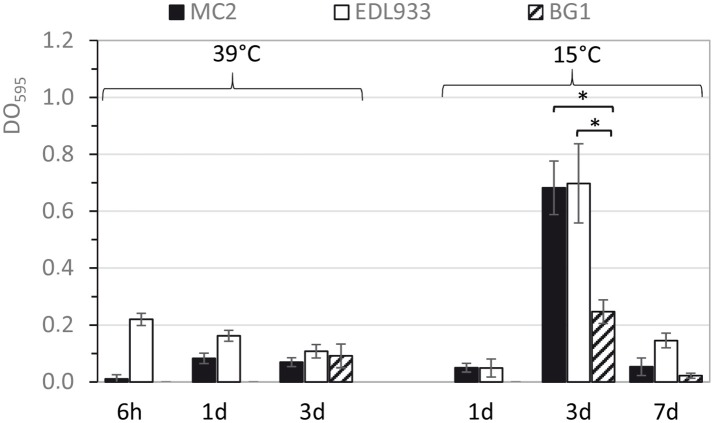
Biofilm formation kinetic of *E. coli* strains on polystyrene surface when incubated in filtered bovine feces. *E. coli* strains were incubated up to 3 and 7 days at 39 and 15°C, respectively. The differences in biofilm formation between the MC2 and BG1 strains were determined by ANOVA with the Tukey *post-hoc* test (^*^p < 0.05).

We analyzed biofilm formation kinetics in filtered bovine feces during 3 and 7 days at 39°C and 15°C respectively. The results showed that *E. coli* MC2 was able to produce strong biofilms in filtered bovine feces at 15°C after 3 days of incubation but was unable to form biofilms at 39°C whatever the time of incubation (Figure 4). Similarly to *E. coli* MC2, EHEC EDL933 also formed strong biofilms in fecal juice at 15°C but was only a weak biofilm producer at 39°C. Interestingly, *E. coli* MC2 was able to form significantly stronger biofilms than commensal *E. coli* BG1 in feces incubated at 15°C at 3 days of incubation (Figure 4). Biofilm formation was also analyzed during incubation of the bacterial strains in M9 minimal media supplemented with glucose (M9-Glc). In contrast to EHEC EDL933, *E. coli* MC2 did not form biofilms at 39°C and weakly produced biofilms (OD_595_ < 0.3) at 15°C during incubation in M9-Glc (data not shown).

### Transcriptomic response of *E. coli* MC2 to stresses in the bovine fecal environment

To investigate the potential factors involved in the persistence of *E. coli* MC2 outside the bovine GIT, the expression change of key stress fitness genes was quantified during incubation of *E. coli* MC2 in bovine feces under conditions mimicking extra-intestinal environment. The genes were selected among the large panel of genes involved in the ability of *E. coli* to survive stresses (nutrient starvation, high osmolarity, fluctuations in temperature, low pH; Supplementary Table 4). Gene expression experiments were performed using RNA collected during the stationary growth phase (7 days of incubation) of *E. coli* MC2, EDL933, and BG1 incubated in filtered bovine feces (Supplementary Figure 3) relative to M9-Glc (see the Materials and Methods section).

**Figure 5:**
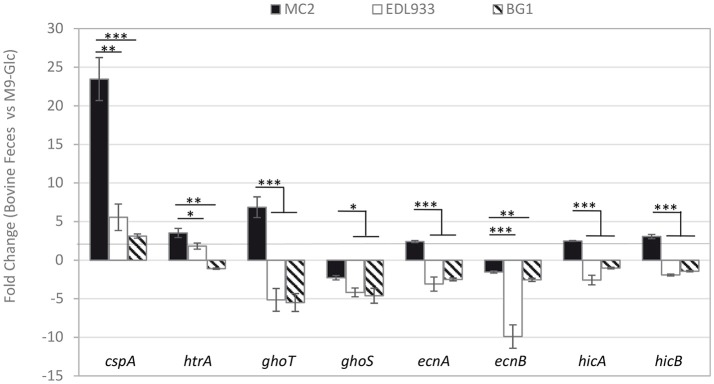
Relative expression levels of stress fitness genes during incubation of *E. coli* O157:H7 MC2 and EDL933, and bovine commensal *E. coli* BG1 in filtered bovine feces in comparison with M9-Glc. Results are average fold changes in expression, with SEM from at least three independent values. ^*^p < 0.05; ^**^p < 0.01; ^***^p < 0.001.

Among the 49 genes tested, *cspA, ghoT, htrA, hicB, hicA*, and *ecnA* were significantly up-regulated in *E. coli* MC2 during its incubation in bovine fecal juice when compared to M9-Glc (fold change >2; *p* < 0.05; Figure 5). Interestingly, the genes *ghoT, hicA* and *ecnA* encoding proteins that are part of the GhoT/GhoS, HicA/HicB or EcnB/EcnA toxin/antitoxin (TA) systems were significantly up-regulated in bovine feces at 15°C. Note that *ghoT* (toxin-encoding gene) but not *ghoS* (antitoxin-encoding genes) was up-regulated in *E. coli* MC2 (Figure 5). As shown in Figure 5, *ecnA* (antitoxin encoding gene) was up-regulated whereas the transcription of *ecnB* (toxin encoding gene) was not induced in *E. coli* MC2.

To broaden these results, the transcription of the stress fitness genes up-regulated in *E. coli* MC2 was also analyzed in *E. coli* EDL933 (EHEC reference strain) and BG1 (bovine commensal strain). Significant differences in gene expression were observed since, in contrast to *E. coli* MC2, (i) the genes *ghoT* and *ecnA* were down-regulated and (ii) the transcription of *htrA, hicA* and *hicB* was not significantly altered in both BG1 and EDL933 *E. coli* strains (Figure 5). Note that (i) similarly to *E. coli* MC2, the gene *cspA* was up-regulated in *E. coli* BG1 and EDL933 but a significant higher fold-change increase was observed in *E. coli* MC2 and (ii) the stress fitness genes expression patterns of *E. coli* BG1 and EDL933 were close to each other and more distant from that of *E. coli* MC2 (Figure 5).

## Discussion

*E. coli* O157:H7 can persist for extended periods in farm environment constituting a risk of cattle contamination. However, bacterial factors and mechanisms that contribute to the persistence of the pathogen outside the bovine intestine remain still poorly understood. With this in mind, we have selected the *E. coli* O157:H7 strain MC2, found to be persistent in farm environment, to attempt to gain insight into mechanisms used by EHEC to transit through the bovine GIT and resist against adverse extra-intestinal conditions. *E. coli* MC2 is potentially pathogen since the bacterium (i) harbored the genes encoding Stx1 and Stx2 toxins (eukaryotic cells apoptosis) and EHEC-hemolysin (lysis of microvascular endothelial cells) and (ii) displayed toxicity to Vero cells and produced A/E lesions. All these characteristics are involved in intestinal damages, bloody diarrhea and/or HUS during human infections. Furthermore, *E. coli* MC2 also possessed the genetic information encoding additional virulence factors closely associated with virulent O157:H7 clinical strains. Similarly to the vast majority of human clinical isolates, *E. coli* MC2 harbored the *tir* 255T allele and the *Q*_933_ anti-terminator variant, a marker of STEC strains with high toxin production (Ahmad and Zurek, 2006; Mellor et al., 2012). The strain MC2 is assigned to the phylo-group D, closely associated with human clinical *E. coli* O157:H7 strains, whereas the phylo-group B1 is prevalent among commensal *E. coli* in the herbivorous hosts (Ziebell et al., 2008; Tenaillon et al., 2010). Importantly, *E. coli* MC2 also possessed the genetic background required for the biosynthesis of flagella (arrival of bacteria to suitable surfaces), type VI secretion system (proteins export) and extracellular matrix (interactions of the bacteria with the surface) involved in biofilm production (Aschtgen et al., 2008; Kapitein and Mogk, 2013; Zhou et al., 2015). Note that the type VI secretion system has also been recently described as contributing to EHEC virulence (Wan et al., 2017). In addition, the type III secretion system required for A/E lesion formation, can also be exploited by the bacteria to inject Nle effectors into human cells, known to counterbalance the inflammatory response of infected enterocytes (Pearson and Hartland, 2014).

A gene polymorphism analysis was conducted as previously described (Eppinger et al., 2011). The results identified *E. coli* MC2 as a strain related to O157:H7 strains involved in human clinical cases. The *E. coli* strain MC2 was found to be a lineage II (LSPA-6 genotype 213111) and clade 7 bacterial strain. Lineage and clade classification of French bovine or human O157:H7 isolates is poorly documented. However, the LSPA-6 genotype 213111 corresponds to a major subtype among cattle and human O157:H7 isolates in Sweden (Söderlund et al., 2014). In Japan, clade 7 isolates are prevalent among clinical *E. coli* O157:H7 (52%) (Ogura et al., 2015). Clade 7 *E. coli* strains are also prevalent among O157:H7 isolates in Australia (70 and 90% of the cattle and human isolates respectively; Mellor et al., 2012). These observations showed that *E. coli* MC2 was related to clinical O157:H7 isolates widespread across several countries and continents. To persist in the farm environment *E. coli* O157:H7 must maintain a dual lifestyle for surviving inside and outside the bovine GIT. In this study, we showed that *E. coli* MC2 survives in rumen fluids and grows in the content of posterior digestive compartments *in vitro*, predicting a successful transit of the bacteria along the bovine digestive tract. The lack of MC2 growth in the rumen contents might be due to specific physiochemical conditions in this compartment (such as high SCFA concentration), nutrients competition with commensal bacteria and/or to the secretion of inhibitory molecules by the ruminal microbiota (Chaucheyras-Durand et al., 2006). Otherwise, *E. coli* MC2 also survived in bovine feces for a long time despite nutritional competition with the large bacterial endogenous population and adverse environmental conditions (low temperature, non-renewal of nutrients). This suggested that the bacteria colonize specific ecological niches and/or express specific mechanisms to resist against nutrient starvation and environmental stresses.

The persistence of *E. coli* O157:H7 in the farm environment is due, at least in part, to its ability to colonize the bovine GIT as well as biotic or abiotic surfaces outside the bovine intestine. Importantly, EHEC strains must produce adhesins to first bring the bacteria closer to the host intestinal epithelia and then establish intimate attachment and produce A/E lesions (McWilliams and Torres, 2014). *E. coli* MC2 possessed the genetic information encoding fourteen adherence systems known to bind to distinct specific receptors. Some of these adhesins possessed properties related to the bovine gut: (i) the F9 fimbriae are involved in calves colonization, (ii) the EhaA and EspP autotransporters adhere *in vitro* to primary bovine rectal epithelial cells, (iii) the HCP pilus is required for the adherence of the bacteria to bovine gut explants, and (iv) FdeC (EaeH) is expressed at 39°C, the internal bovine temperature, but not at 37°C (Dziva et al., 2004; Easton et al., 2014; McWilliams and Torres, 2014). Furthermore, the *Q*_933_ anti-terminator involved in *stx2* regulation, is probably indirectly involved in bovine intestinal colonization since the production of Stx2 (not cytotoxic to bovine enterocytes) enhances *E. coli* O157:H7 colonization of cattle intestinal tissues (Baines et al., 2008). In addition, the tip protein FliD included in H7 flagella, mediates the adhesion of *E. coli* O157:H7 to bovine rectal epithelium (Mahajan et al., 2009). Noteworthy, EhaB, ELF, HCP, and UpaG bind to laminin, an extracellular matrix present in the mammalian intestine that promotes bacterial adhesion to host tissues (Simon-Assmann et al., 2010; McWilliams and Torres, 2014). In addition to these adherence systems, the metalloprotease StcE could play an important role in bacterial colonization of the bovine gut. Indeed, StcE, only secreted by pathogenic *E. coli*, is required to cleave the protein backbone of the mucin glycoproteins recovering the host enterocytes and contributes to efficient intimate adherence of the bacteria to host epithelium (Hews et al., 2017). The large number of colonization factors that bind to distinct host receptors reflects the capacity of *E. coli* MC2 to colonize different segments of the bovine GIT. Iron acquisition, essential for bacterial growth, plays also an important role in *E. coli* gut colonization. *E. coli* MC2 has the potentiality to scavenge iron by the mean of siderophore enterobactin and to cleave cyclic enterobactin into linear enterobactin by the mean of the Fes esterase, thus allowing the release of iron into the bacterial cytoplasm (Braun, 2003). In addition, the ChuA haem-binding receptor is synthesized by the bacteria in response to iron limitation and the Hma receptor promotes haemin uptake independently to ChuA (Torres and Payne, 1997; Hagan and Mobley, 2009). The redundancy of genes encoding haem-receptors in the MC2 genome suggests an important role of haemin and/or hemoglobin as iron source. As described above, the MC2 genome shared the genes encoding the production of EHEC-hemolysin that is able to lyse red blood cells and increase hemoglobin concentration *in vivo*. In fact, the capacity of *E. coli* to produce hemolysis coupled with the ability to transport haem from hemoglobin, result in an efficient iron acquisition mechanism for pathogens responsible for hemolytic lesions. As a comparison, the genome of the commensal strain BG1 only shared the genetic information coding for enterobactin (Segura et al., 2017).

Phenotype characteristics can also be considered as biomarkers to identify differences in *E. coli* O157:H7 environmental persistence (Mukherjee et al., 2008; Franz et al., 2011). In the present study, the vast majority of the adherence systems encoded by the MC2 genome is involved in the early stages of biofilm formation. Biofilm production, known to protect attached bacteria from desiccation, UV radiation and other environmental stresses, is considered as a survival strategy used by the bacteria in hostile environments (Puttamreddy et al., 2010; Yaron and Römling, 2014; Vogeleer et al., 2015). For example, the operons *csgDEFG* and *csgBAC* encoded the synthesis of curli fimbriae that are required by *E. coli* O157:H7 for adhesion to biotic and abiotic surfaces and biofilm formation under conditions encountered in extra-intestinal environments (e.g., low temperature, nutrient limitation; Sharma and Bearson, 2013). In this study, *in vitro* experiments showed that *E. coli* MC2 is a strong biofilm producer when incubated in filtered fecal samples but only a weak biofilm producer in minimal media. Furthermore, the highest production of biofilm was observed at a temperature corresponding to extra-intestinal environments. Importantly, *E. coli* MC2 had a greater ability to form biofilms in bovine feces than bovine commensal *E. coli* suggesting that EHEC O157:H7 are potentially more efficient than bovine commensal *E. coli* to survive in cow manure.

Interestingly, Vanaja et al. compared the expression pattern of *E. coli* O157:H7 strains with genotypes that predominate among bovine isolates (bovine-biased genotypes) and human clinical cases (clinical genotypes) and showed that genes involved in the resistance to adverse environments are up-regulated in the bovine-biased genotype (Vanaja et al., 2010). In this study, we showed for the first time that an O157:H7 *E. coli* strain responded to temperature stresses by inducing the genes *cspA* and *htrA* during its survival in bovine feces at low temperature. HtrA is a bifunctional protein with protease and chaperone properties at high and low temperatures, respectively. These two properties are required to refold or degrade misfolded proteins that accumulate in the periplasm under stressful conditions (Spiess et al., 1999; Skorko-Glonek et al., 2008). The RNA chaperone CspA is required for preventing the formation of secondary structures in mRNA molecules at low temperature (Ivancic et al., 2013). Interestingly, *cspA* is expressed transiently in the early stage of cold shock acclimation of the bacteria incubated in laboratory medium (Ivancic et al., 2013) whereas the gene was induced in *E. coli* MC2 after 7 days of incubation at low temperature. Furthermore, the transcription of the genes *cspA* and *htrA* was not induced in the bovine commensal *E. coli* BG1 and the reference EHEC strain EDL933 suggesting that the persistent bovine *E. coli* MC2 exhibits specific responses to cold stress in bovine feces at low temperature.

*E. coli* MC2 also activated GhoT/GhoS, EcnB/EcnA, and HicA/HicB toxin/antitoxin (TA) systems in bovine feces at low temperature. These TA systems are composed of a toxin, which causes bacterial growth arrest and dormancy during environmental stresses, and a cognate antitoxin, which neutralizes the toxin activity during normal growth conditions (for a review see Page and Peti, 2016). Such TA systems are known to act as a bacteriolytic module involved in programmed bacterial cell death (EcnB/EcnA) and to increase bacterial persistence in response to amino acid or glucose starvation, (HicA/HicB) or chemical stresses (GhoT/GhoS) (Bishop et al., 1998; Jørgensen et al., 2009; Cheng et al., 2014). In addition, GhoT not only leads to the formation of dormant cells under stress conditions but also impacts early biofilm formation by *E. coli* (Wang et al., 2013). TA systems activation is very complex and can occur at both transcriptional and post-transcriptional levels (Otsuka, 2016; Page and Peti, 2016; Turnbull and Gerdes, 2017). For example, nutrient starvation induced *hicAB* transcription but toxin HicA also stimulates the synthesis of antitoxin HicB when HicA is in excess (Turnbull and Gerdes, 2017). Nevertheless, our q-PCR data strongly suggest a potential role for TA systems in the survival of O157:H7 *E. coli* outside the animal GIT. Interestingly, our results also showed that significant differences in stress fitness genes expression was observed not only between O157:H7 *E. coli* and commensal *E. coli* bovine strains (MC2 and BG1 respectively) but also between O157:H7 *E. coli* strains (MC2 and EDL933). This suggested that *E. coli* strains with different origins respond differently to stresses highlighting the potential role of cold shock proteins and TA systems in the persistence of bovine O157:H7 *E. coli* outside the bovine gut. Taken together, our results strongly suggested that *E. coli* MC2 must combat low temperature, nutrients starvation and chemical stresses to survive in bovine feces at low temperature. Activation of TA systems by the bacteria constitutes new areas of work to attempt to explain the persistence of O157:H7 *E. coli* in hostile extra intestinal environments. Further studies will be required to attribute an unequivocal role of GhoT/GhoS, EcnB/EcnA and HicA/HicB TA systems in the persistence of STEC O157:H7 in bovine excrement.

In conclusion, *E. coli* MC2 possessed many characteristics common among clinical O157:H7 *E. coli* strains and harbored a large arsenal of virulence determinants involved in different aspects of *E. coli* O157:H7 pathogenesis. The large number of colonization factors known to bind to intestinal epithelium and to biotic and abiotic surfaces, the capacity of *E. coli* MC2 to produce biofilms and to activate stress fitness genes in bovine feces could explain the persistence of the bacteria in the farm environment.

## Author contributions

YB, AS, DB, HB, and EF conceived and designed the experiments. AS, MB, MK, AD, and DB performed the experiments. PA and YB performed the bioinformatics analysis. YB, AS, PA, GJ, and EF analyzed the data and wrote the manuscript. All authors have read and approved the final manuscript.

### Conflict of interest statement

The authors declare that the research was conducted in the absence of any commercial or financial relationships that could be construed as a potential conflict of interest.

## Abbreviations

A/E: attaching and effacing
CFU: colony-forming unit
DC: digestive content
EHEC: enterohaemorrhagic *E. coli*
GIT: gastro-intestinal tract
SCFA: Short chain fatty acid
STEC: Shiga toxin-producing *E. coli*.
