# Supplementary material for: Factors Involved in the Persistence of a Shiga Toxin-Producing Escherichia coli O157:H7 Strain in Bovine Feces and Gastro-Intestinal Content
Source: Front Microbiol. 2018 Mar 9;9:375. doi: 10.3389/fmicb.2018.00375 (PMC5854682; doi:10.3389/fmicb.2018.00375)
Supplement: Supplementary file 1 [file Table1.DOCX]

Supplementary Material

**FACTORS INVOLVED IN THE PERSISTENCE OF BOVINE *ESCHERICHIA COLI* O157:H7 IN THE FARM ENVIRONMENT**

**Audrey Segura, Pauline Auffret, Delphine Bibbal, Marine Bertoni, Alexandra Durand, Grégory Jubelin, Monique Kérourédan, Hubert Brugère, Yolande Bertin, Evelyne Forano***

*** Correspondence:** Evelyne Forano : evelyne.forano@inra.fr

# Supplementary Figures and Tables

- 1. **Supplementary Figures**

**Supplementary Figure 1.** Cytotoxic activity of MC2 on Vero cells. The survival rate of Vero cells treated with *E. coli* DH5α cell culture supernatant was defined as 100%. EDL933 was used as a positive control.

**Supplementary Figure 2.** Formation of A/E lesions after infection of Hela cells by *E. coli* MC2, by using the FAS test. The EPEC strain E2348/69 and *E. coli* DH5α were used as a positive and negative controls, respectively.

**Supplementary Figure 3.** Growth curves of *E. coli* strains MC2, BG1 and EDL933 in filtered bovine fecal juice. The cultures were incubated at 15°C without shaking. Each time point is the mean of three independent experiments.

- 1. **Supplementary Tables**

**Supplementary Table 1.** Bacterial strains used in phylogenetic analysis.

| Strain | Number | Origin | Source | Clade | Lineage |  | Reference |
| --- | --- | --- | --- | --- | --- | --- | --- |
| 08BKT061141 | NZ_JJOL00000000.1 | bovine feces | cattle | 7 | II |  | Soderlung et al., 2014 |
| 09BKT002497 | JJOJ00000000.1 | bovine feces | cattle | 5 6 | I/II |  | Soderlung et al., 2014 |
| 09BKT048303 | NZ_JJOM00000000.1 | bovine feces | cattle | 7 | II |  | Soderlung et al., 2014 |
| 287Jul8F | NZ_LHAJ00000000.1 | bovine feces | cattle (low shedder) | 2 | I* |  | Munns et al., 2016 |
| 299Jul8F | NZ_LHAK00000000.1 | bovine feces | cattle (low shedder) | 2 | I* |  | Munns et al., 2016 |
| 342Jul26F | NZ_LHAL00000000.1 | bovine feces | cattle (low shedder) | 5 | II* |  | Munns et al., 2016 |
| 7.1_Anguil | LAZD00000000.1 | farm | cattle | 6 | I/II* |  | Amigo et al., 2015 |
| EC4042 | ABHM00000000.2 | spinach outbreak | human patient | 8** | I/II |  | Cote et al., 2015 |
| EC4076 | ABHQ00000000.1 | spinach outbreak | human patient | 8** | I/II |  | Cote et al., 2015 |
| EC4113 | ABHP00000000.1 | spinach outbreak | human patient | 8** | I/II |  | Cote et al., 2015 |
| EC4115 | CP001164.1 | spinach outbreak | human patient | 8 | I/II |  | Cote et al., 2015 |
| EC4206 | ABHK00000000.2 | spinach outbreak | human patient | 8** | I/II |  | Cote et al., 2015 |
| EC869 | ABHU00000000.1 | ground beef | cattle | 7 | II |  | Eppinger et al., 2011 |
| EDL933 | AE005174.2 | hamburger outbreak | human patient | 3 | I |  | Latif et al., 2014 |
| FRIK2000 | ACXO00000000.1 | bovine reservoir | cattle | 7 | II |  | Dowd et al., 2010 |
| FRIK966 | ACXN00000000.1 | bovine reservoir | cattle | 7 | II |  | Dowd et al., 2010 |
| JEONG-1266 | NZ_CP014314.1 | bovine feces | cattle (super shedder) | 8** | I/II* |  | Teng et al., 2016 |
| K3_66F | NZ_LHAN00000000.1 | bovine feces | cattle (super shedder) | 2 | I* |  | Munns et al., 2016 |
| K9_45F | LHAP00000000.1 | bovine feces | cattle (super shedder) | 2 | I* |  | Munns et al., 2016 |
| MC2 | NJDB00000000.1 | bovine feces | cattle | 7** | II* |  | This study |
| Rafaela_II | LAYW00000000.1 | farm | cattle | 8 | I/II* |  | Amigo et al., 2015 |
| Sakai | NC_002695.1 | radish sprouts outbreak | human patient | 1 | I |  | Hayashi et al., 2001 |
| SS17 | NZ_CP008805.1 | bovine feces | cattle (super shedder) | 8 | I/II |  | Cote et al., 2015 |
| SS52 | NZ_CP010304.1 | bovine feces | cattle (super shedder) | 8** | I/II |  | Katani et al., 2015 |
| TW14359 | NC_013008.1 | spinach outbreak | human patient | 8 | I/II |  | Strachan et al., 2015 |
| T1543_06 | JJOK00000000.1 | bovine feces | cattle | 8 | I/II |  | Soderlung et al., 2014 |

* Lineage determined in this study using the LSPA-6 method (see the Materials and Methods section).

** Clade determined in this study using the Manning SNP typing method (see the Materials and Methods section).

Amigo, N., Mercado, E., Bentancor, A., Singh, P, Vilte, D., Gerhardt, E., et al. (2015). [Clade 8 and Clade 6 strains of *Escherichia coli* O157:H7 from cattle in Argentina have hypervirulent-like phenotypes](https://www.ncbi.nlm.nih.gov/pmc/articles/PMC4452545/). *PLoS One.*  10(6): e0127710.

Cote, R., Katani, R., Moreau, M.R., Kudva, I.T., Arthur, T.M., DebRoy, C., et al. (2015). [Comparative analysis of super-shedder strains of *Escherichia coli* O157:H7 reveals distinctive genomic features and a strongly aggregative adherent phenotype on bovine rectoanal junction squamous epithelial cells.](https://www.ncbi.nlm.nih.gov/pubmed/25664460) *PLoS One.* 10(2):e0116743.

[Dowd, S.E](https://www.ncbi.nlm.nih.gov/pubmed/?term=Dowd%20SE%5BAuthor%5D&cauthor=true&cauthor_uid=20156085)., [Crippen, T.L](https://www.ncbi.nlm.nih.gov/pubmed/?term=Crippen%20TL%5BAuthor%5D&cauthor=true&cauthor_uid=20156085)., [Sun, Y](https://www.ncbi.nlm.nih.gov/pubmed/?term=Sun%20Y%5BAuthor%5D&cauthor=true&cauthor_uid=20156085)., [Gontcharova, V](https://www.ncbi.nlm.nih.gov/pubmed/?term=Gontcharova%20V%5BAuthor%5D&cauthor=true&cauthor_uid=20156085)., [Youn, E](https://www.ncbi.nlm.nih.gov/pubmed/?term=Youn%20E%5BAuthor%5D&cauthor=true&cauthor_uid=20156085)., [Muthaiyan, A](https://www.ncbi.nlm.nih.gov/pubmed/?term=Muthaiyan%20A%5BAuthor%5D&cauthor=true&cauthor_uid=20156085)., et al. (2010). Microarray analysis and draft genomes of two *Escherichia coli* O157:H7 lineage II cattle isolates FRIK966 and FRIK2000 investigating lack of Shiga toxin expression. [*Foodborne Pathog Dis.*](https://www.ncbi.nlm.nih.gov/pubmed/?term=FRIK2000) 7, 763-73.

Eppinger, M., Mammel, M.K., Leclerc, J.E., Ravel, J., and Cebula, T.A. (2011). [Genome signatures of *Escherichia coli* O157:H7 isolates from the bovine host reservoir.](https://www.ncbi.nlm.nih.gov/pubmed/21421787) *Appl Environ Microbiol.*  77, 2916-2925.

Hayashi, T., Makino, K., Ohnishi, M., Kurokawa, K., Ishii, K., Yokoyama, K., et al. (2001). [Complete genome sequence of enterohemorrhagic *Escherichia coli*O157:H7 and genomic comparison with a laboratory strain K-12.](https://www.ncbi.nlm.nih.gov/pubmed/11258796) *DNA Res.* 8, 11-22.

[Katani, R](https://www.ncbi.nlm.nih.gov/pubmed/?term=Katani%20R%5BAuthor%5D&cauthor=true&cauthor_uid=28797098)., [Cote, R](https://www.ncbi.nlm.nih.gov/pubmed/?term=Cote%20R%5BAuthor%5D&cauthor=true&cauthor_uid=28797098)., [Kudva, I.T](https://www.ncbi.nlm.nih.gov/pubmed/?term=Kudva%20IT%5BAuthor%5D&cauthor=true&cauthor_uid=28797098)., [DebRoy, C](https://www.ncbi.nlm.nih.gov/pubmed/?term=DebRoy%20C%5BAuthor%5D&cauthor=true&cauthor_uid=28797098)., [Arthur, T.M](https://www.ncbi.nlm.nih.gov/pubmed/?term=Arthur%20TM%5BAuthor%5D&cauthor=true&cauthor_uid=28797098)., and [Kapur, V](https://www.ncbi.nlm.nih.gov/pubmed/?term=Kapur%20V%5BAuthor%5D&cauthor=true&cauthor_uid=28797098). (2017). Comparative genomics of two super-shedder isolates of *Escherichia coli* O157:H7. *[PLoS One.](https://www.ncbi.nlm.nih.gov/pubmed/28797098)* 12(8):e0182940.

Munns, K.D., Zaheer, R., Xu, Y., Stanford, K., Laing, C.R., Gannon, V.P., et al. (2016). [Comparative genomic analysis of](https://www.ncbi.nlm.nih.gov/pubmed/27018858) *[Escherichia coli](https://www.ncbi.nlm.nih.gov/pubmed/27018858)* [O157:H7 isolated from Super-Shedder and Low-Shedder cattle.](https://www.ncbi.nlm.nih.gov/pubmed/27018858) *PLoS One*. 11(3):e0151673.

Söderlund, R., Jernberg, C., Ivarsson, S., Hedenström, I., Eriksson, E., Bongcam-Rudloff, E. et al. (2014). [Molecular typing of *Escherichia coli*O157:H7 isolates from Swedish cattle and human cases: population dynamics and virulence.](https://www.ncbi.nlm.nih.gov/pubmed/25143581) *J Clin Microbiol.* 52, 3906-3912.

[Strachan, N.J](https://www.ncbi.nlm.nih.gov/pubmed/?term=Strachan%20NJ%5BAuthor%5D&cauthor=true&cauthor_uid=26442781)., [Rotariu, O](https://www.ncbi.nlm.nih.gov/pubmed/?term=Rotariu%20O%5BAuthor%5D&cauthor=true&cauthor_uid=26442781)., [Lopes, B](https://www.ncbi.nlm.nih.gov/pubmed/?term=Lopes%20B%5BAuthor%5D&cauthor=true&cauthor_uid=26442781)., [MacRae, M](https://www.ncbi.nlm.nih.gov/pubmed/?term=MacRae%20M%5BAuthor%5D&cauthor=true&cauthor_uid=26442781)., [Fairley, S](https://www.ncbi.nlm.nih.gov/pubmed/?term=Fairley%20S%5BAuthor%5D&cauthor=true&cauthor_uid=26442781)., [Laing, C](https://www.ncbi.nlm.nih.gov/pubmed/?term=Laing%20C%5BAuthor%5D&cauthor=true&cauthor_uid=26442781)., et al. (2015). Whole genome sequencing demonstrates that geographic variation of *Escherichia coli* O157 genotypes dominates host association. *[Sci Rep.](https://www.ncbi.nlm.nih.gov/pubmed/?term=escherichia+coli+O157+Strachan+2015)*5:14145.

[Teng, L](https://www.ncbi.nlm.nih.gov/pubmed/?term=Teng%20L%5BAuthor%5D&cauthor=true&cauthor_uid=27056233)., [Ginn, A](https://www.ncbi.nlm.nih.gov/pubmed/?term=Ginn%20A%5BAuthor%5D&cauthor=true&cauthor_uid=27056233)., [Jeon, S](https://www.ncbi.nlm.nih.gov/pubmed/?term=Jeon%20S%5BAuthor%5D&cauthor=true&cauthor_uid=27056233)., [Kang, M](https://www.ncbi.nlm.nih.gov/pubmed/?term=Kang%20M%5BAuthor%5D&cauthor=true&cauthor_uid=27056233)., and [Jeong, K.C](https://www.ncbi.nlm.nih.gov/pubmed/?term=Jeong%20KC%5BAuthor%5D&cauthor=true&cauthor_uid=27056233). (2016). Complete genome sequence of an *Escherichia coli* O157:H7 strain isolated from a Super-Shedder steer. *[Genome Announc.](https://www.ncbi.nlm.nih.gov/pubmed/27056233)*  4(2). pii: e00258-16.

**Supplementary Table 2.** Oligonucleotide primers used in this study (relative RT-qPCR)

| Gene | Primer sequence |  |
| --- | --- | --- |
|  | Forward | Reverse |
| *acrA* | GCAGCTTAGCCCTAACAGGA | CCGCTAACTTGAGGACGAAC |
| *ahpC* | GACGTTGCTGACCACTACGA | CGCATGTTGTCGAAGTTACG |
| *bolA* | CAACCCGTATTCCTCGAAGT | CAGCGCATGAACGGTAGTAG |
| *chaB* | CACGTTCTACCGTCTCATGC | CCCTTTGGCATATTCATGCT |
| *cspA* | CCGGTAAAATGACTGGTATCG | TCGATGGTGAAGGACACTTTC |
| *cspD* | CCAAAGGGTTTGGTTTCATC | ACTTCGACGGGCACAATAAC |
| *clpA* | CACCACGACGTGCGTTATAC | CGCCACATTAACGGTTTTCT |
| *dps* | GTAGCTCTGGGGACCACTCA | AGGATATCTGCGGTGTCGTC |
| *ecnA* | ATGATGAAACGCCTTATC | GGCAGCGCGGGAGATGG |
| *ecnB* | GGTGAAGAAGACAATTGCAG | TTATTGCTGCGCTTTCGTTG |
| *elaB* | CACGTATCGATGACGACCTG | CTGCTTCGCCCGATAGTAAT |
| *gabD* | CGGTGGGTAATGAACTGACC | GCTTTATCGAGATCGGCATC |
| *gadA* | CTGGGGCCGTATGAGTTCA | GGGTGTATCCCGGATCTTC |
| *gadE* | GAGAAATTAGATGCCGAGAG | TTGTGAATTCTTATGGGGCA |
| *ghoS* | GGAAGGTAAAAACAAGTTC | CGATGTCGTTATTGCGAAG |
| *ghoT* | GGCACTATTCTCTAAAAT | AAGTAATGCCACAGGCAGAC |
| *grpE* | CATGGATCAGCACGAAGAGA | AGGTTTTCCATTTCGGCTTT |
| *hchA* | GCCGCAGAAAGATGAAAAAG | AGCTACGTCCTGGCTTTCAG |
| *hdeB* | CACTGGTGAACGCACAATCT | GGAATTTGAGTGAGATCGGTTT |
| *hicA* | AAAGCGAGTTCAGACGTTGG | CCGAGTTGTTTCAGGATTGC |
| *hicB* | GGATATCCCTGAAGCGTTGA | GCGACGCTCAAAGGTACTTC |
| *htrA* | CAGGTTGATTCCAGCTCCAT | TCAGCGATGTTTTTCACAGC |
| *hyaA* | TATGGTCAGCGTATCCACGA | AAGAAACGCCATCATTCCAG |
| *katE* | GGCTAAGTCAGACGCCATTC | TTCAGCTGGTCGTCAGTCAG |
| *mazE* | TAAAGCGTTGGGGAAATTCA | TCGTTGACCAGTTCAGCAAG |
| *mazF* | TTGACCCGACAAAAGGTAGC | AGCTAACGCTACGCCATCAC |
| *mdtE/yhiU* | CACCTTTACAGGCCGAGCTA | GGTGACATTGGCTTCTGCTT |
| *osmC* | AGAAAGGTCAGGCACACTGG | AAAGCGCCATTGAGAAACAT |
| *osmY* | GAAGGCTCTGTGAAGGGCTA | GCTTTGGCGATACTTTCAGC |
| *otsB* | CAGAACCGTTAACCGAAACC | GCCCTGATATCAATGCCAAT |
| *poxB* | CTGCCGGTGAAAATTATCGT | GGCTTCATCGATTTCAGAGG |
| *psiF* | TTGCTTTTCGGTCTGGTTTT | TGCTGTTCTTCAGGCAATCA |
| *relE* | CCCCTCCAACAAGGAAAGAG | TTGCTAAAGGCCAGAAATGC |
| *rpoS* | AAGAGAACGGTCCGGAAGAT | GAGGCCAATTTCACGACCTA |
| *slp* | GCGAAGCCTGATATTGAAGC | GGATGCCCTGCATATTCACT |
| *speA* | GAACGTATGGCGGACAAAAT | CGATAGCACCGTCAGAGTCA |
| *sspA* | ATGAGTTCAGCCTGGTCGAT | GCATTTCACGTTCTGCTTCA |
| *sugE* | TTCAACCATCAGCTTTGCAG | GGTAATAACACTCGGCGTCAA |
| *tufA* | TGGTTGATGACGAAGAGCTG | GCTCTGGTTCCGGAATGTAG |
| *uspA* | CTATGGCTCGCCCATACAAT | GTGATTGGGTAGCCTGCATT |
| *yciE* | GCAGCACATTAGCGAAACAA | AGCCACTGATCGAACCTTTG |
| *yehX* | GCTGGTCACGCATGATATTG | CGCCACACTACGTAACGAAA |
| *yehY* | AAGAATACGCCAACCGTCAG | GAAGGCACGGTCTGAATGAC |
| *ygiW/gadW* | GCATCAGCACTGTTTCAGGA | GACATCCTGGAGGCGAGTAA |
| *yggE* | GCGCTACCATGTTTCCAACT | CAGGTGTTTTAGCGGGTTGT |
| *yhiO* | TGCGATCCATTGCTCTATCA | CAATGCGCTGGTCAGAATAA |
| *yhiU* | AATGTCACCTCGCCGATTAC | CACTGGCGACCTCTTCTTTC |
| *yhiV/cmeB* | CCAATGGTATGTACGCAACG | CGACCAGGTCTGCCATAAAT |

**Supplementary Table 3** VFs encoding genes in the MC2 genome. The MC2 genome was screened for virulence factors using blastn and the Virulence Factors database (VFDB) DNA dataset.

Excel file data sheet 1

**Supplementary Table 4**. Stress fitness genes tested in this study (relative RT-qPCR)

| Gene | Protein or function |
| --- | --- |
| *acrA* | Multidurg efflux pomp subunit (antibiotic and drug resistance) |
| *ahpC* | Alkyl hydroperoxide reductase subunit C (oxydative stress) |
| *bolA* | Protein BolA (carbon starvation; heat shock; osmotic, oxidative and acid stresses) |
| *chaB** | Cation transport regulator (salinity stress) |
| *clpA** | Chaperone ClpA, ATP-binding component of serine protease (degradation of abnormal protein) |
| *cspA* | Cold shock protein CspA (temperature stress) |
| *cspD** | Cold shock protein CspD (temperature stress) |
| *dps** | DNA binding protein DpS (nutritional deprivation; oxydative stress) |
| *ecnA* | Antidote lipoprotein EcnA (programmed cells death under starvation conditions) (TA system) |
| *ecnB* | Bacteriolytic lipoprotein EcnB (programmed cells death under starvation conditions) (TA system) |
| *elaB* | Membrane protein ElaB (multiple stress response) |
| *gabD* | Succinate-semialdehyde dehydrogenase (putrescine metabolism) |
| *gadA* | Glutamate decarboxylase (acid and osmotic stresses; anaerobic phosphate starvation) |
| *gadE* | Transcriptional activator GadE (acid stress; pH homeostasis; anaerobic phophate starvation) |
| *ghoS* | Antitoxin GhoS (chemical stresses) (TA system) |
| *ghoT* | Toxin GhoT (chemical stresses) (TA system) |
| *grpE* | Nucleotide exchange factors GrpE (heat and hyperosmotic shocks) |
| *hchA* | Hsp31 Chaperone periplasmic protein (acid stress; heat shock; starvation; osmotic stress) |
| *hdeB* | Periplasmic chaperone HdeB (acid stress) |
| *hicA* | Toxin HicA (amino acid and glucose starvation) (TA system) |
| *hicB* | Antitoxin HicB (amino acid and glucose starvation) (TA system) |
| *htrA* | Protease HtrA (temperature and oxydative stressess) |
| *hyaA* | Uptake hydrogenase small subunit precursor (starvation) |
| *katE* | Catalase (peroxide dammage; oxydative and osmotic stresses) |
| *mazE* | Antitoxin MazE (high temperatures; DNA damage; oxidative stress) (TA system) |
| *mazF* | Toxin MazF (high temperatures; DNA damage; oxidative stress) (TA system) |
| *mdtE* | Multidrug resistance subunit MdtE (nitrosative dammages) |
| *osmC** | Protein OsmC (osmotic stress) |
| *osmY** | Protein OsmY (osmotic stress) |
| *otsB* | Trehalose-6-phosphate phosphatase (smotic stress; sucrose starch) |
| *poxB* | Pyruvate oxidase (osmotic stress; phosphate starvation ) |
| *psiF* | Protein Psi (phosphate starvation) |
| *relE* | Toxin RelE (amino acid; glucose and nitrogen starvation) |
| *rpoS* | σ38 subunit RNA Polymerase RpoS (general stress response) |
| *slp* | Lipoprotein Slp (carbon starvation; phosphorus limitation) |
| *speA* | Arginine decarboxylase (acid resistance) |
| *sspA* | Stringent starvation protein A (acid resistance) |
| *sugE* | Protein SugE (resistance to quaternary ammonium compound; multidrug efflux pump) |
| *terE* | Protein TerE (tellurium resistance) |
| *uspA** | Protein UspA (universal stress response) |
| *yciE* | YciE/YciF family protein (osmotic stress) |
| *yehX* | ABC transporter ATP-binding subunit YehX (osmotic stress) |
| *yehY* | Transporter permease protein YehY (osmotic stress) |
| *ygiW/gadW* | Protein YgiW (GadW) (hydrogen peroxide acid stress) |
| *yggE* | Protein YggE (oxydative stress; response to tellurite toxicity) |
| *yhiO* | Protein YhiO (glucose, nitrogen and phosphate stravation; osmotic and oxidative stresses) |
| *yhiU* | Proteins YhiU (acid stress; multidrug efflux pump) |
| *yhiV/cmeB* | Inner membrane transporter CmeB (acid stress; multidrug efflux pump) |

* Stress fitness genes preferentially expressed in *E. coli* O157:H7 strains with the bovine-biased genotype (Vanaja et al. (2010)
